# Editorial: Therapy resistance in tumor microenvironment: metabolic reprogramming and purinergic signaling

**DOI:** 10.3389/fonc.2023.1297152

**Published:** 2023-11-06

**Authors:** Michele Zanoni, Henrique Borges da Silva, Elena Adinolfi

**Affiliations:** ^1^ Biosciences Laboratory, IRCCS Istituto Romagnolo per lo Studio dei Tumori (IRST) “Dino Amadori”, Meldola, Italy; ^2^ Department of Immunology, Mayo Clinic, Scottsdale, AZ, United States; ^3^ Department of Medical Sciences, Section of Experimental Medicine, University of Ferrara, Ferrara, Italy

**Keywords:** cancer, metabolism, tumor microenvironment, purinergic signaling, metastasis, resistance to therapy, stemness

Fundamental pathophysiological processes such as tissue homeostasis, neurodegeneration, immunity, inflammation, and cancer are modulated by purinergic signaling (1). The tumor microenvironment (TME) is enriched in nucleosides and nucleotides, and its cellular and biochemical composition affects tumor progression, metastatic spread, and response to therapy (2). Current therapeutic approaches such as surgery, chemotherapy, targeted therapy, hormonal therapy, radiotherapy, and immunotherapy can profoundly alter TME composition, causing extensive cell death, activating the immune system, and provoking a massive release of inflammatory and damage-associated molecular patterns (DAMPS), including nucleotides (ATP and ADP) and nucleosides (adenosine) (3). The impact of these purines on TME strictly depends on the repertoire of P1 (also known as A receptors or ADORA) and P2 receptors and ectonucleotidases (CD39 and CD73) expressed by tumor, immune, and stromal cells, affecting cancer proliferation and several metabolic pathways and promoting both immunostimulatory (eATP) or immunosuppressive (adenosine) responses (2, 4). This Research Topic includes two reviews and three research articles that discuss the role of purinergic signaling in the context of tumor progression and resistance to therapies, also proposing the purinergic axis as a novel therapeutic target in cancer.

In this Research Topic, Kaur and Dora made an overview of the functional role of purinergic signaling in cancer, focusing on several key aspects of oncological diseases. ATP and adenosine (ADO) play a central role in the overall configuration of the tumor milieu, and their levels in the tumor interstitium are mainly controlled by the balance between their secretion/release and degradation. ATP is the main source for the increase in ADO concentration in TME due to the sequential activity of CD39 and CD73 ectonucleotidases. ATP and ADO regulate several cellular functions, including intracellular energy homeostasis, nucleotide synthesis, immune response, cell proliferation, epithelial-to-mesenchymal transition (EMT), angiogenesis, metabolism, and migration in an autocrine/paracrine way by activating purinergic receptors P2 and P1, respectively. Among P2 receptors, the P2X7 receptor (P2X7R) has been extensively associated with cancer pathogenesis, exerting a dual function based on the degree of activation. When stimulated at a tonic level, P2X7R promotes tumor growth and cell proliferation, enhancing oxidative phosphorylation (OXPHOS) efficiency and promoting aerobic glycolysis through the upregulation of the expression of the plasma membrane glucose transporter GLUT1 and of other enzymes of the glycolytic pathway thus increasing ATP and lactate generation (5). Moreover, the signaling cascade activated by P2X7 involves changes in intracellular Ca^2+^ homeostasis, activating the PI3K/AKT and ERK/MAPK pathways. Alternatively, prolonged stimulation causes the opening of a large macropore in the membrane that triggers cell death (6). Understanding the intracellular mechanisms that govern P2X7R expression could be relevant, representing an important biological and clinical issue for several cancers. Benito-León et al. showed that inhibiting DUSP1 phosphatase enhances the expression of P2X7 receptor in N2a neuroblastoma cells by activating the p38 signaling pathway. This mechanism could be of particular relevance in TME, where the activation of p38 in response to inflammatory signals and environmental stresses upregulate P2X7R expression, which, in turn, may promote cancer cell proliferation, energy production, migration, and invasiveness of cancer cells. In addition to the mechanism regulating P2X7R expression and P2X7R’s two-faced behavior, the numerous splice variants of the *p2rx7* gene also play an important role in determining the different cellular responses to eATP. Among these, the human P2X7B and the full-length P2X7A isoforms are the most well-characterized and studied in cancer. B isoform is truncated in the C-terminal region and retains the ion channel activity but lacks the ability to form the pro-apoptotic macropore. As the full-length isoform, P2X7B promotes tumor growth and invasiveness, metastatic dissemination, and resistance to therapy (7). Here, Arnaud-Sampaio et al. demonstrate that P2X7B is involved in neuroblastoma chemoresistance while the A isoform exerts a complementary and opposite role. B isoform promotes resistance to retinoids, maintaining cancer cells in a stem-like phenotype, inducing EMT, impairing autophagy, and triggering drug efflux via MRP-type transporters. On the contrary, P2X7A has an essential role in macropore-dependent autophagy-mediated cell death and is important for cell differentiation to a more epithelial-prone phenotype. In addition, A isoform favors drug response, also downregulating drug efflux pump expression. Beyond P2X7R, other members of the purinergic axis are reported to participate in resistance to different anti-cancer therapies, as reported in the review by Zanoni et al. Conventional treatments such as chemotherapy and radiotherapy induce cell death and release of ATP from dying cells in the TME, re-activating the immune response through immunogenic cell death (ICD). However, both P2XRs (as P2X7R) and P2YRs as P2Y1, P2Y2, P2Y6, and P2Y12 expressed by cancer cells confer resistance to cytotoxic drugs and potentiate the cellular response to DNA damage induced by RT, being responsible for tumor recurrence. Furthermore, eATP released in the TME after treatment is quickly hydrolyzed by CD39 and CD73 ectonucleotidases expressed by both cancer and immune cells, exerting a potent immunosuppressive activity by acting on the ADORAs. These data open new interesting scenarios in combining traditional anti-cancer interventions with therapies targeting the purinergic axis. In particular, the combination of approved immunomodulators with adenosinergic targeting drugs was demonstrated to improve the efficacy of immunotherapies in several preclinical models. Moreover, a clinical trial in patients with refractory renal cell cancer using A2AR antagonist in combination with anti-PD-L1 has provided clinical benefit to the patients, shaping the immune system through an increased recruitment of CD8^+^ T cells into the tumor mass. In line with that and to deepen the complex interaction between the different members of the purinergic family, in this Research Topic De Marchi et al. have demonstrated that tumors growing in the absence of host P2X7R are characterized by the upregulation of A2AR, VEGF, and TGF-β promoting the establishment of an immunosuppressive TME enriched in T regulatory cells (8), a reduction of pro-inflammatory cytokines levels and an increased neovascularization. Therefore, this novel crosstalk between P2X7R and A2AR can be of great interest as targeting both receptors may represent a more effective therapeutic strategy in cancer.

In summary, this Research Topic offers a comprehensive overview of how the purinergic signaling regulates tumor growth and immune responses in the TME. The input from these studies will be crucial, in our opinion, to outline future research to define how these purinergic sensors affect antitumor immunity and cancer cell homeostasis in different therapeutic contexts.

## Author contributions

MZ: Writing – original draft, Writing – review & editing. HS: Writing – original draft, Writing – review & editing. EA: Writing – original draft, Writing – review & editing.
